# Walking for Well-Being: Are Group Walks in Certain Types of Natural Environments Better for Well-Being than Group Walks in Urban Environments?

**DOI:** 10.3390/ijerph10115603

**Published:** 2013-10-29

**Authors:** Melissa R. Marselle, Katherine N. Irvine, Sara L. Warber

**Affiliations:** 1 Institute of Energy and Sustainable Development, De Montfort University, Queens Building, the Gateway, Leicester LE1 9BH, UK; E-Mail: kirvine@dmu.ac.uk; 2 The James Hutton Institute, Craigiebuckler, Aberdeen AB15 8QH, UK; E-Mail: katherine.irvine@hutton.ac.uk; 3 University of Michigan Integrative Medicine, Department of Family Medicine, University of Michigan, 1018 Fuller Street, Ann Arbor, MI 48104, USA; E-Mail: swarber@umich.edu

**Keywords:** natural environment, green space, well-being, Walking for Health, walking group, walking, green exercise, England, UK

## Abstract

The benefits of walking in natural environments for well-being are increasingly understood. However, less well known are the impacts different types of natural environments have on psychological and emotional well-being. This cross-sectional study investigated whether group walks in specific types of natural environments were associated with greater psychological and emotional well-being compared to group walks in urban environments. Individuals who frequently attended a walking group once a week or more (*n* = 708) were surveyed on mental well-being (Warwick Edinburgh Mental Well-being Scale), depression (Major Depressive Inventory), perceived stress (Perceived Stress Scale) and emotional well-being (Positive and Negative Affect Schedule). Compared to group walks in urban environments, group walks in farmland were significantly associated with less perceived stress and negative affect, and greater mental well-being. Group walks in green corridors were significantly associated with less perceived stress and negative affect. There were no significant differences between the effect of any environment types on depression or positive affect. Outdoor walking group programs could be endorsed through “green prescriptions” to improve psychological and emotional well-being, as well as physical activity.

## 1. Introduction

Well-being is fundamental for health. The World Health Organization defines health as “a state of complete physical, mental and social well-being and not merely the absence of disease or infirmity” [1]. Yet, globally cardiovascular disease (CVD), obesity and depression are projected to increase, threatening both physical and mental well-being [2,3]. To reduce the burden on healthcare systems, prevention of these chronic conditions is essential [3,4].

Walking has been shown to be a cost-effective and accessible form of physical exercise that can prevent CVD [2,5], address obesity [6,7], and reduce symptoms of depression [3,8,9]. Walking is the most popular form of physical activity in the United States and the United Kingdom [10,11] yet less than half of adults in both countries meet the recommended levels of physical activity [12,13].

The social and physical environment can influence whether a person takes up walking [14]. People are more likely to walk in the company of another person or a pet [15]; this is particularly relevant for women [16]. Individuals are more likely to walk in physical environments that are aesthetically beautiful and maintained [15,17,18,19], accessible [17], contain footpaths [18,19,20] and that are perceived as safe [19,21]. There is a positive association between natural environments and physical activity [22]; natural environments are also more likely to be used for physical activity than recreation centers or sports facilities [20].

### 1.1. Group Walking

Group walking outdoors is an integration of both the social and physical environment correlates of walking. The Centers for Disease Control and Prevention [11] recommends walking in a group to increase walking behavior, as the social environment of the walking group may augment adherence to walking [23]. Research has shown that people both prefer [24] and enjoy [25] walking with others outdoors, more than walking outdoors alone. Less well researched, however, are the well-being effects from group walks in natural environments.

### 1.2. Benefits from Walking in Natural Environments

The well-being effects of natural environments have been extensively reviewed [26,27,28,29,30]. Two main theories provide a framework for understanding how natural environments enhance well-being. The first, Attention Restoration Theory (ART), posits natural environments contain stimuli that allow for the restoration from mental fatigue, which is the depletion of one’s ability to direct attention [31,32]. The stimuli in natural environments are hypothesized to effortlessly attract one’s involuntary attention, allowing for the restoration of one’s directed attention [32]. The second theory is the psycho-physiological stress reduction framework which posits that nature initiates innate emotional, physiological, cognitive and behavioral responses [33,34]. According to the theory, the restorative benefits of nature are reduced negative affect and physiological arousal, and enhanced positive affect and attention [33,34]. These theoretical benefits of the natural environment are exclusive of those experienced through physical activity. For example, viewing the natural environment can result in the restorative benefits for attention and emotional well-being [35,36,37,38,39].

Walking in natural environments has been found to provide additional benefits to emotional well-being compared to walking indoors [30,40] or in an urban environment [30]. Walking alone in natural environments has been shown to enhance psychological and emotional well-being by improving attention [41,42] and positive emotions [41,42,43,44], and by reducing negative emotions [41,42,45] and stress [41] when compared to walking alone in urban environments.

Group walks in natural environments can have an effect on well-being greater than the act of walking or the social environment. A group walk in the natural environment has been shown to significantly improve emotions and self-esteem when compared to a group walk indoors [46]. Self-esteem was significantly greater when walking in a group in the natural environment compared to a sedentary social group [47]. Sugiyama *et al.* [48] found the “greenness” of the environment was strongly associated with mental health, over and above the effects of walking and social coherence. The social and environmental context together may moderate the effect of emotional well-being. For example, walking with others may enhance the experience of being in an urban environment but decrease the experience of a natural environment on indicators of well-being [24,49]*.*

### 1.3. Walking in Different Types of Natural Environments

Studies of walking and well-being have, to date, investigated a limited set of natural environments. Parks and university campuses are the most common types of natural environments in walking studies [30,40]. None of the walking studies reviewed by Bowler *et al.* [30] and Thompson Coon *et al.* [40] examined more than one type of natural environment. Further research has been called for in order to investigate the contribution of different types of natural environments on well-being [17,28,30,40,50,51,52].

Several recent studies have specifically considered this issue. Three studies focused on the impact of physical exercise in different environments on mental and emotional well-being. Walking alone in a maintained forest had a greater increase on positive affect and greater decrease in negative affect than a walk in a wild, unmaintained forest [53]. Running indoors whilst viewing scenes of either a pleasant urban or rural environment had a greater effect on self-esteem over and above the effect of exercise alone [54]. Exercising in parks and woodland environments was associated with decreased risk of mental ill health and exercising in parks and outdoor sports fields was associated with greater mental well-being when compared to not exercising in these respective environment settings [52]. One secondary data analysis study investigated the impact of both the broad and specific types of environments on recalled feelings of restoration [55]. Compared to rural green space (*i.e.*, the countryside), urban green space was associated with significantly less restoration, but coastal environments were associated with greater restoration [55]. With regard to specific environment types, beach, coastal, forest/woodland, hill/mountain and farmland environments were all significantly positively associated with restoration, compared to the countryside environments [55]. Two epidemiological-type studies have found positive associations between different types of natural environments and physical and mental health. Perceived health was positively associated with the amount of farmland, woodland or grassland around one’s home [56]. Mental health was positively associated with the amount of farmland near one’s home [57].

Aquatic environments have also been shown to have a greater effect on health and well-being than other natural environments. A “blue” gradient in health and mental health has been found, in which self-reported health [58] and mental health [58,59] increased the closer an individual lived to the sea, over and above the effects of green environments. Exercising near waterside environments demonstrated greater improvements in self-esteem and mood compared to exercising in urban green space, farmland and woodland environments [60]. Beach and river environments are experienced with high levels of psychological well-being and low levels of negative feelings [61].

The perceived degree of naturalness or level of biodiversity of the natural environment has also been found to contribute to well-being [51,62,63]. Hinds and Sparks [61] found “more” natural environments (e.g., mountain, forest, woodland, valley) were associated with greater psychological well-being, than “less” natural environments (e.g., parks, gardens, farmland fields). Psychological well-being was positively associated with the actual number of plant species and habitat types in urban green space [62] and perceived number of plant, bird and butterfly species in riparian green space [63].

### 1.4. Study Focus

The study reported here is a sub-study from a larger observational, longitudinal panel study about the psychological and emotional well-being of individuals who do and do not take part in outdoor walking groups. This sub-study specifically sought to examine the contribution of six different types of natural environments for a group walk on psychological and emotional well-being. Following recommendations from Bowler *et al.* [30], this study considers whether there is an “added benefit” (p. 2) from a specific type of natural environment by investigating the difference in well-being between group walkers in a specific type of natural environment compared to group walkers in urban environments. Any differences between the two groups may then be attributable to the environment.

The research questions were:

1. Does environment type contribute to psychological and emotional well-being, after statistical adjustment for socio-demographics, walking behavior, physical activity, and stressful life events?
2. Which types of natural environments for a group walk are associated with psychological and emotional well-being, compared to urban environments?

## 2. Method

A cross-sectional design investigated the psychological and emotional well-being of individuals who participated in a Walking for Health (WfH) walking group in 2011. Walking for Health (www.walkingforhealth.org.uk) is one of the largest public health interventions for physical activity in the UK [64]. The program supports walking groups throughout England by providing free, organized, led group walks. Currently, 70,000 people regularly attend 3,400 WfH walks provided by 600 walk schemes [65]. All WfH group walks take place outdoors in England. As each walking group has the liberty to choose the location of the walk, WfH walks occur in a variety of outdoor environments.

### 2.1. Participants

1,258 WfH group walkers participated in the larger study. A subsample of 708 frequent WfH walkers—individuals from the larger sample who had attended a WfH walking group at least once a week during the previous 13 weeks, and who had no missing data—were analyzed for this study. The sample was female (62%), aged 55 years or older (92%), university educated (46%), married, civil partnered or cohabiting (72%) and lived in the least deprived areas of England (51%). Table 2 further details the demographics of the sample.

### 2.2. Measures

#### 2.2.1. Outcome Variables

Five outcome measures of psychological and emotional well-being were assessed.

Mental well-being was measured using the 14-item Warwick Edinburgh Mental Well-being Scale (WEMWBS) [66] which assesses both hedonic (positive emotion, satisfaction) and eudiamonic (functioning) aspects of positive mental health [66]. The WEMWBS is “suitable for use in measuring mental well-being at a population level” [66] (p. 10), and has been recommended for use by the UK government [67]. Participants rated each statement in relation to their experience in the past two weeks on a 5-point scale (1 = none of the time; 5 = all of the time), resulting in a minimum score of 14 and maximum score of 70. A higher score indicates higher level of mental well-being. This measure has been used in previous nature and health studies in the UK [52,68,69].

Depression was measured with the 10-item Major Depressive Inventory (MDI) [70]. The MDI assesses depression according to the Diagnostic and Statistical Manual (DSM) and the International Classification of Diseases 10 symptoms of moderate to severe depression (ICD-10) [71]. The MDI can be scored as either a diagnostic instrument or as a general depression rating scale [70]. The general depression rating scale was used here. Participants rated how frequently they felt a certain way in the past two weeks on a 6-point scale (0 = at no time; 5 = all the time), resulting in a total score range from 0 (no depression) to 50 (extreme depression) [70]. The MDI has been used with the general population in Denmark [70,72] and Sweden [73]. The measure has been used in the UK on particular populations [74,75]. This is the first time the measure has been used in nature and health research.

The 10-item Perceived Stress Scale (PSS) [76] was used to measure perceptions of stress. Participants were asked to rate the frequency of experiencing certain thoughts and feelings in the past two weeks on a 5-point scale (0 = never; 4 = very often). Total scores range from 0 to 40; higher scores indicate greater psychological stress. This measure has been used in previous nature and health studies [69,77] and in the UK [69].

The Positive and Negative Affect Schedule (PANAS) [78] was used to measure both positive and negative affect. Participants rated the frequency of experiencing 10 positive and 10 negative emotions in the past two weeks on a 5-point scale (1 = very slightly or not at all; 5 = extremely). For each scale, total scores range from 10 to 50 with higher scores demonstrating greater positive or negative affect. The PANAS has been used in previous nature and health studies [42,79,80] and with the general UK population [81].

#### 2.2.2. Predictor Variables

Demographic characteristics included gender (0 = male, 1 = female), age (0 = 18–54, 1 = 55 and over) [64,82], ethnicity (0 = white, 1 = non-white), marital status (0 = single, widowed, divorced, 1 = married, civil partnered, cohabitating) [83], highest level of education (1= no education, 2 = secondary education, 3 = tertiary education) [84,85] and tertile of the English Index of Multiple Deprivation 2010 overall rank (1 = most deprived, 3 = least deprived) [86].

Data on the frequency and duration of WfH walks and non-group walks as well as physical activity were also collected. Given the selected subsample for this study (*i.e.*, frequent WfH walkers), WfH walk frequency data demonstrated a limited range and positively skewed distribution. Transformations were unsuccessful, thus this variable was dichotomized for analysis (0 = WfH walk once a week, 1 = WfH walk more than once a week) [87]. Duration of WfH walks was assessed in 15-min. increments (range 15–195 min). Due to positive skewness, this variable was log-transformed. Participants were also asked for the frequency and duration they walked in green space without their walking group. Frequency of non-group walks in green space was collected on a 7-point scale (1 = never; 7 = daily). Duration of non-group walks in green space was assessed in 15-min. increments (range 0–195 min). Physical activity was assessed by asking participants: “In the last seven days on how many days have you done a total of 30 min or more of physical activity, which was enough to raise your breathing rate?” [88,89]. Participants were asked to include their WfH walks as well as any other walking, cycling, sports or exercise. Responses were recorded on an 8-point scale (0 = 0 days; 7 = 7 days).

Recent stressful life events that might have occurred in the previous 13 weeks were assessed with the List of Threatening Experiences [90,91]. The sum of 11 stressful life events was calculated, resulting in a range from 0 to 11 stressful life events; the resultant variable was log-transformed due to positive skewness.

#### 2.2.3. Walk Environment Type

The most common type of environment in which the participant walked with a WfH group during the 13-week study period was assessed with the question: “What is the main type of environment you walk in with this [WfH] group?” Participants selected one response from a list of 10 categories. Response options were drawn from the WfH Walk Route Assessment questionnaire [92], which itself matched the environment types outlined in the English Planning Policy Guidance (PPG) 17 [93] (pp. 13–14). For this study, the “cemeteries, disused churchyards and other burial grounds” category was excluded and a “coastal environments” category [94] was included into the list of walk environment types.

Response distribution across the ten provided environment type categories was unequal: natural and semi-natural places (24.8%); green corridor (26.8%); farmland (13.3%); parks and gardens (6.8%); urban public space (5.8%); coastal (6.1%); amenity green space (2.3%); allotments, community gardens, urban farms (0.4%), outdoor sports facilities (0.1%) or an “other” write-in category (10%). For analysis purposes, four of the categories were combined following PPG 17 definitions of environment types into a new walk environment category entitled “Urban green space” (see Table 1). Coastal and urban public space environments were considered empirically and theoretically important [30,58] and were left intact. Write-in responses were analyzed and recoded into an existing environment type category, where appropriate. Forty write-in responses that described a combination of two or more environment types (e.g., “a combination of all above”; “a mixture of urban public space and green corridor”; “the walks cover farmland, green corridor and coastal”) were coded into a new category entitled “Mixture”. Table 1 provides details for the frequency with which each environment type was selected following recategorization of original responses for the subsample.

**Table 1:** The number of frequently attending Walking for Health (WfH) walkers as a function of the main type of environment in which walked with their WfH group (*n* = 708).

Type of environment	Example provided in questionnaire	*Frequency* *n* (%)
Natural and semi-natural places	Country park, nature reserve	216 (30.5%)
Green corridor	River path, cycleways, bridleways	190 (26.8%)
Farmland	*No example given*	102 (14.4%)
Urban green space ^a^	Public gardens, formal parks, amenity green space, allotments, community gardens, urban farms, outdoor sports pitches	71 (10.0%)
Coastal	Seaside, estuary	45 (6.4%)
Urban public space	Streets, shopping centers, plaza	44 (6.2%)
Mixture ^b^	“A combination of all of the above”	40 (5.6%)

^a^ New category analyzed by the authors combines four original categories: parks and gardens; allotments, community gardens and urban farms; amenity green space; and outdoor sports pitches. ^b^ New category analyzed by the authors; category contains “other” write-in responses from participants that described two or more different environment types. Example not provided in the questionnaire, but a participant response to the “other” write in category.

### 2.3. Procedure

Participants were recruited from the WfH participant database. Contact details were obtained through the WfH program. The supplied sampling frame consisted of all individuals involved in WfH group walks who had given consent and provided an email address to be contacted for program evaluation purposes. To capture individuals who had recently participated in WfH, eligibility criteria was limited to individuals who had attended at least one WfH walk 6 months before the start of the study on 1 August 2011 [95].

Participants were invited to take part in the larger research study via an invitation e-mail which contained a weblink to an online questionnaire. Thirteen weeks later, all participants who completed the first questionnaire (Time 1) were invited by email to complete a second online questionnaire (Time 2). Here we draw on cross-sectional data from Time 2 data only; Time 2 data were collected between 15 November 2011 and 1 December 2011. A prize draw of 150 British Pounds worth of shopping vouchers was provided as incentive for participation. All study materials were piloted and underwent ethics review by De Montfort University’s Human Research Ethics committee. Participants gave informed consent prior to starting the Time 1 online questionnaire. Individuals were able to withdraw from the research study by using an “Unsubscribe” weblink provided in all study documents (*i.e.*, invitation and reminder emails, first page of the online questionnaire).

### 2.4. Statistical Analysis

Differences on socio-demographics, walking behavior, physical activity and recent stressful life events between participants across the seven walk environment types were examined using chi-square and one-way ANOVA with Bonferroni *post-hoc* tests (Bonferroni corrected α-level (0.05/7) = 0.007). Regression analyses investigated the contribution of each walk environment type to mental well-being, depression, perceived stress, and positive and negative affect, holding significant covariates constant. Separate regression analyses were run for each outcome variable. Dummy variables were created for each walk environment type. The reference group was “urban public space”. This analysis enabled the comparison of the change in the outcome variable as a participant changes from WfH group walks in urban public space to WfH group walks in a more “natural” environment.

First, backwards stepwise regression was used to identify a subset of non-environment related predictor variables that significantly predicted each outcome variable (Step 1). Predictor variables entered into the backwards stepwise regression included: gender, age, ethnicity, marital status, education, deprivation, frequency of WfH group walks and non-group walks in green space, log-transformed duration of WfH walks, duration of non-group walks, frequency of physical activity in the past week and log-transformed recent stressful life events. Residual plots from the regression models were analyzed to determine how closely these followed a normal distribution. Where residuals showed large deviations from normality, transformations were applied to the outcome variables; a log transformation was conducted for both negative affect and depression [87].

Second, hierarchical multiple regression was conducted to examine the relationship between environment type for a WfH walk and psychological and emotional wellbeing while holding constant identified significant predictors from the backwards stepwise regression. All analyses were performed using SPSS 20.0 software. Variables identified as significant predictors in the backwards stepwise regression were entered in the first block (Step 1). WfH walk environment type dummy variables were entered in the second block (Step 2). Significance levels for all regression analyses were set at *p* < 0.05.

## 3. Results

### 3.1. Predictor Variables by Environment Type

Characteristics for participant groups across the seven walk environment types are provided in Table 2 and Table 3. Table 2 details the socio-demographic characteristics. Groups differed significantly on ethnicity (χ^2^ (6) = 14.40, *p* = 0.03), age (χ^2^ (6) = 33.70, *p* < 0.001) and deprivation (χ^2^ (12) = 27.37, *p* = 0.01). More participants of a non-white ethnicity attended WfH walks in urban green space (11.3%) than any other type of environment. One quarter (25%) of frequent WfH walkers in urban public space were aged 18–54, more than any other environment type. More participants from the most deprived areas of England would frequently attend WfH walks in urban green space (21.1%) than any other environment. Over half (55.6%) of all frequent group walkers in a coastal environment lived in moderately deprived areas of England. Approximately 60% of WfH walkers in green corridor environments lived in the least deprived areas in England.

**Table 2:** Socio-demographic characteristics of frequently attending group walkers as a function of the main type of environment in which they walked with the group in the past 13 weeks (*n* = 708).

Variable	Natural and semi-natural (*n* = 216)	Green corridor (*n* = 190)	Farmland (*n* = 102)	Urban green space (*n* = 71)	Coastal (*n* = 45)	Urban public space (*n* =44)	Mixture (*n* = 40)
Female *%* (*n*)	66.2 (143)	60.5 (115)	50.0 (51)	64.8 (46)	64.4 (29)	56.8 (25)	70.0 (28)
White *%* (*n*) *	95.4 (206)	97.9 (186)	98.0 (100)	**88.7 (63)**	97.8 (44)	97.7 (43)	97.5 (39)
Aged 55+ *%* (*n*) ***	92.6 (200)	92.1 (175)	99.0 (101)	81.7 (58)	95.6 (43)	**75.0 (33)**	95.0 (38)
Married, civil partnered, cohabitating % (n)	66.7 (144)	75.8 (144)	82.4 (84)	64.8 (46)	68.9 (31)	70.5 (31)	67.5 (27)
**Education % (n)**							
No educations	8.8 (19)	9.5 (18)	5.9 (6)	5.6 (4)	6.7 (3)	9.1 (4)	7.5 (3)
Secondary education	49.1 (106)	43.7 (83)	39.2 (40)	45.1 (32)	57.8 (26)	45.5 (20)	50.0 (20)
Tertiary education	42.1 (91)	46.8 (89)	54.9 (56)	49.3 (35)	35.6 (16)	45.5 (20)	42.5 (17)
Deprivation % (n) **							
Most deprived	11.6 (25)	11.6 (22)	6.9 (7)	**21.1 (15)**	13.3 (6)	13.6 (6)	10.0 (4)
Moderate deprived	35.2 (76)	29.5 (56)	39.2 (40)	43.7 (31)	**55.6 (25)**	34.1 (15)	47.5 (19)
Least deprived	53.2 (115)	**58.9 (112)**	53.9 (55)	35.2 (25)	31.1 (14)	51.3 (23)	42.5 (17)

Note: All analyses were Pearson Chi-square. Bold text indicates category that differs from the rest. * *p* < 0.05. ** *p* < 0.01. *** *p* < 0.001.

Table 3 provides details on additional characteristics of interest. Groups significantly differed on duration of WfH walks (*F* (6, 701) = 13.74, *p* < 0.001) and frequency of non-group walks in green space (*F* (6, 701) = 3.23, *p* = 0.004). Bonferroni *post-hoc* tests indicated that WfH walks in urban green space and urban public space were of significantly less duration compared to WfH walks in natural environments, green corridor, and farmland (*p* < 0.007 for all); additionally, WfH walks in urban public space were of significantly less duration than WfH walks in coastal and mixture environments (*p* < 0.007 for all). Bonferroni *post-hoc* tests indicated a significant difference on the frequency of non-group walks between group walkers in green corridor and urban green space (mean difference = 0.81, 95% CI = 0.20, 1.42, *p* < 0.007). Group walkers in green corridor environments took the most non-group walks (*M* = 4.13) whilst group walkers in urban green space took the fewest (*M* = 3.32).

**Table 3:** Frequency and duration of WfH group and non-group walks, physical activity, and number of recent stressful life events of frequently attending WfH group walkers as a function of the main type of environment in which they walked with the group in the past 13 weeks (*n* = 708).

Variable	Natural and semi-natural (*n* = 216)	Green corridor (*n* = 190)	Farmland (*n* = 102)	Urban green space (*n* = 71)	Coastal (*n* = 45)	Urban public space (*n* =44)	Mixture (*n* = 40)
WfH walk at least once a week % (*n*)	70.4 (152)	71.1 (135)	75.5 (77)	64.8 (46)	75.6 (34)	77.3 (34)	60.0 (24)
Duration of a WfH walk (in minutes) M (SD) ^^,^***	96 (45)	94 (40)	100 (41)	**74 (45)**	88 (41)	**61 (32)**	83 (38)
Frequency of non-group walks M (SD) **	3.90 (1.47)	**4.13 (1.33)**	3.84 (1.40)	**3.32 (1.54)**	3.96 (1.68)	3.52 (1.47)	3.98 (1.44)
Duration of non-group walks (in minutes) M (SD)	93 (56)	95 (50)	91 (52)	79 (59)	88 (54)	89 (57)	103 (57)
Physical activity (days) M (SD)	3.50 (1.73)	3.66 (1.73)	3.51 (1.72)	3.69 (1.85)	4.00 (1.73)	3.57 (1.55)	3.78 (1.83)
Recent stressful life events ^ M (SD)	0.52 (0.85)	0.62 (.85)	0.61 (.96)	0.79 (1.01)	0.73 (0.92)	0.52 (.85)	0.58 (0.84)

Note: All analyses were one-way ANOVA Bonferroni *post-hoc* tests; bold text indicates group difference. Higher scores indicate greater: duration of WfH walks (range 15–195 min); frequency of non-group walks (1 = never; 7 = daily); duration of non-group walks in green space (range 0–195 min); physical activity (0 = 0 days; 7 = 7 days); and number of stressful life events experienced in the past 13 weeks (range 0–11). ^ = log-transformed variable for ANOVA analyses. Non-transformed means and standard deviations presented here. ** *p* < 0.01. *** *p* < 0.001.

### 3.2. Well-Being Outcomes

Table 4 shows the means and standard deviations for psychological and emotional well-being of WfH walkers who frequently attended a WfH walk in each type of environment.

**Table 4:** Mean and standard deviation for mental well-being, depression, perceived stress, positive affect and negative affect of frequent WfH walkers within each environment type (*n* = 708).

Environment type	MentalWell-being		Depression		Perceived Stress		Positive Affect		Negative Affect		
	*M*	*SD*	*M*	*SD*	*M*	*SD*	*M*	*SD*	*M*	*SD*	*n*
Natural and semi-natural	53.17	7.30	6.06	5.26	11.17	6.12	34.80	7.45	14.06	4.33	216
Green corridor	54.23	7.16	5.68	5.02	9.93	5.55	35.99	6.31	13.65	4.09	190
Farmland	54.64	6.83	5.60	5.95	9.57	5.66	36.34	6.04	12.68	3.30	102
Urban green space	52.68	9.63	7.93	7.48	12.56	7.74	34.97	7.96	15.54	6.04	71
Coastal	52.42	6.86	5.96	4.02	12.16	5.94	34.49	6.93	15.42	4.99	45
Urban public space	51.61	7.62	8.09	7.37	13.23	5.91	34.14	7.37	15.57	5.75	44
Mixture	53.10	6.41	7.48	6.31	12.63	6.30	35.58	6.55	14.70	5.11	40

Note: Non-transformed means and standard deviations for depression and negative affect presented here. Higher scores indicate greater: mental well-being (range 14–70); depression (range 0–50); perceived stress (range 0–40); positive affect (range 10–50); and negative affect (range 10–50).

#### 3.2.1. Mental Well-Being and Environment Type

Marital status and physical activity accounted for 3.2% of the variance of mental well-being in the initial model and remained significantly positively associated with mental well-being in the final model (β = 0.13, *p* < 0.001; β = 0.11, *p* = 0.003, respectively; see Table 5). The final model remained significant although the addition of walk environment type accounted for a non-significant increase of the variance explained (∆R^2^ = 0.011, *p* = 0.247). Of the six walk environment predictors, participants who frequently attended WfH group walks in farmland environments (β = 0.13, *p* = 0.04) were significantly associated with greater mental well-being in comparison with participants who frequently attended WfH group walks in urban public spaces. The effect of WfH group walks in green corridor environments was marginally significant (β = 0.15, *p* = 0.05).

**Table 5:** Hierarchical regression analyses for variables predicting mental well-being of frequent group walkers attending at least once a week (*n* = 708).

Variables	Mental Well-Being				*F*	*df*	*p*
	*B*	*SE B*	β	*p*			
Step 1					11.574	2, 705	<0.001
Constant	50.12	0.77		<0.001			
Marital Status	2.34	0.61	0.14	<0.001			
Physical Activity	0.46	0.16	0.11	0.004			
Step 2					3.890	8, 699	<0.001
Constant	48.39	1.31		<0.001			
Marital Status	2.18	0.62	0.13	<0.001			
Physical Activity	0.47	0.16	0.11	0.003			
Natural & Semi-Natural	1.67	1.21	0.10	0.17			
Green corridor	2.46	1.22	0.15	0.05			
Farmland	2.79	1.32	0.13	004			
Urban Green Space	1.13	1.40	0.05	0.42			
Coastal	0.64	1.55	0.02	0.68			
Mixture of 2 or more	1.45	1.60	0.05	0.36			

Note: Step 1: R^2^ = 0.032, Adj R^2^ = 0.029, *p* < 0.001. Step 2: R^2^ = 0.043, Adj R^2^ = 0.032, *p* < 0.001; ∆R^2^ = 0.011 (*p* = 0.247). *B* = regression coefficient, β = standardized regression coefficient. Marital Status: Reference category was single, divorced, widowed. Physical Activity: 0 = 0 days; 7 = 7 days. Environment type: Reference category was Urban Public Space.

#### 3.2.2. Depression and Environment Type

In the initial model, age, marital status, physical activity, recent stressful life events and duration of WfH walks accounted for a significant 9.5% of the variance of log-transformed depression (Table 6). The final model with all predictors was also significant. In Step 2, marital status (β = −0.12, *p* = 0.002) and physical activity (β = −0.16, *p* < 0.001) were significantly negatively associated, and recent stressful life events (β = 0.19, *p* < 0.001) was significantly positively associated with depression. A marginal significant association was found between age (β = −0.07) and duration of WfH group walks (β = −0.08) with a reduction in depression (*p* = 0.05). Environment type accounted for a non-significant increase of the variance explained by the model (∆R^2^ = 0.007, *p* = 0.499); there were no significant differences between WfH group walks in urban public space and WfH group walks in any type of natural environment on log-transformed depression.

**Table 6:** Hierarchical regression analyses for variables predicting log-transformed depression of frequent group walkers attending at least once a week (*n* = 708).

Variables	Depression ^				*F*	*df*	*p*
	*B*	*SE B*	β	*p*			
Step 1					14.699	5, 702	<0.001
Constant	1.27	0.12		<0.001			
Age	−0.09	0.04	−0.08	0.02			
Marital Status	−0.09	0.03	−0.12	0.001			
Physical Activity	−0.03	0.01	−0.16	<0.001			
Recent stressful life events ^	0.30	0.06	0.19	<0.001			
Duration of WfH walks ^	−0.17	0.06	−0.10	0.01			
Step 2					7.162	11, 696	<0.001
Constant	1.24	0.13		<0.001			
Age	−0.08	0.04	−0.07	0.05			
Marital Status	−0.08	0.03	−0.12	0.002			
Physical Activity	−0.03	0.01	−0.16	<0.001			
Recent stressful life events ^	0.30	0.06	0.19	<0.001			
Duration of WfH walks ^	−0.13	0.06	−0.08	0.05			
Natural & Semi-Natural	−0.07	0.05	−0.10	0.18			
Green corridor	−0.08	0.05	−0.12	0.11			
Farmland	−0.09	0.06	−0.10	0.12			
Urban Green Space	−0.03	0.06	−0.03	0.65			
Coastal	−0.05	0.07	−0.04	0.41			
Mixture of 2 or more	−0.01	0.07	−0.01	0.89			

Note: Step 1: R^2^ = 0.095, Adj R^2^ = 0.088, *p* < 0.001. Step 2: R^2^ = 0.102, Adj R^2^ = 0.087, *p* < 0.001; ∆R^2^ = 0.007 (*p* = 0.499). *B* = regression coefficient, β = standardized regression coefficient. Age: Reference category was 18–54 years of age. Marital Status: Reference category was single, divorced, widowed. Physical Activity: 0 = 0 days; 7 = 7 days. Recent stressful life events range was 0–11 stressful events. Duration of WfH walks, in minutes, range was 15 min to 195 min (3 h 15 min) (original, untransformed variable range). Environment type: Reference category was Urban Public Space. ^ = log-transformed variable.

#### 3.2.3. Perceived Stress and Environment Type

Age, marital status, recent stressful life events and frequency of non-group walks in green space accounted for a significant 8.8% of the variance of perceived stress in the initial model (Table 7). In the final model age, marital status and frequency of non-group walks in green space were significantly negatively associated with perceived stress, and recent stressful life events was significantly positively associated with perceived stress. Walk environment type was an additional explanatory variable accounting for a significant increase of the variance explained by the model (R^2^ = 0.112, Adj R^2^ = 0.099, *p* < 0.001; ∆R^2^ = 0.025, *p* = 0.004). Of the six environment types, participants who frequently attended WfH group walks in green corridor (β = −0.20, *p* = 0.005) and farmland environments (β = −0.17, *p* = 0.006) were associated with significantly less perceived stress in comparison with participants who frequently attended WfH walks in urban public space.

**Table 7:** Hierarchical regression analyses for variables predicting perceived stress of frequent group walkers attending at least once a week (*n* = 708).

Variables	Perceived Stress				*F*	*df*	*p*
	*B*	*SE B*	β	*p*			
Step 1					16.881	4, 703	<0.001
Constant	14.91	1.04		<0.001			
Age	−2.73	0.80	−0.12	0.001			
Marital Status	−1.41	0.49	−0.10	0.004			
Recent stressful life events ^	7.02	1.11	0.23	<0.001			
Frequency non-group walks	−0.38	0.15	−0.09	0.014			
Step 2					8.807	10, 697	<0.001
Constant	15.96	1.26		<0.001			
Age	−2.35	0.81	−0.11	0.004			
Marital Status	−1.19	0.49	−0.09	0.02			
Recent stressful life events ^	7.03	1.11	0.23	<0.001			
Frequency of non-group walks	−0.32	0.15	−0.08	0.04			
Natural & Semi-Natural	−1.57	0.98	−0.12	0.11			
Green corridor	−2.81	0.99	−0.20	0.005			
Farmland	−2.96	1.08	−0.17	0.006			
Urban Green Space	−1.05	1.13	−0.05	0.35			
Coastal	−0.83	1.26	−0.03	0.51			
Mixture of 2 or more	−0.11	1.29	−0.004	0.93			

Note: Step 1: R^2^ = 0.088, Adj R^2^ = 0.082, *p* < 0.001. Step 2: R^2^ = 0.112, Adj R^2^ = 0.099, *p* < 0.001; ∆R^2^ = 0.025 (*p* = 0.004). *B* = regression coefficient, β = standardized regression coefficient. Age: Reference category was 18–54 years of age. Marital Status: Reference category was single, divorced, widowed. Recent stressful life events range was 0–11 stressful events. Frequency of non-group walks: 1 = never; 7 = daily. Environment type: Reference category was Urban Public Space. ^ = log-transformed variable.

#### 3.2.4. Positive Affect and Environment Type

Table 8 provides results for positive affect. In Step 1, marital status, physical activity and duration of non-group walks in green space accounted for a significant 5.6% of the variance of positive affect. The final model with all predictors was significant. In Step 2, marital status (β = 0.08, *p* = 0.03), physical activity (β = 0.17, *p* < 0.001) and duration of non-group walks in green space (β = 0.11, *p* = 0.004) were significantly positively associated with positive affect. Environment type accounted for a non-significant increase of the explained variance (∆R^2^ = 0.008, *p* = 0.403); there were no significant differences between the effect of WfH group walks in urban public space and WfH group walks in any type of natural environment on positive affect.

**Table 8:** Hierarchical regression analyses for variables predicting positive affect for frequent group walkers attending at least once a week (*n* = 708).

Variables	Positive Affect				*F*	*df*	*p*
	*B*	*SE B*	β	*p*			
Step 1					13.874	3, 704	<0.001
Constant	30.62	0.78		<0.001			
Marital Status	1.43	0.57	0.09	0.01			
Physical Activity	0.67	0.15	0.17	<0.001			
Duration of non-group walks	0.01	0.01	0.11	0.003			
Step 2					5.315	9, 698	<0.001
Constant	29.60	1.25		<0.001			
Marital Status	1.28	0.57	0.08	0.03			
Physical Activity	0.68	0.15	0.17	<0.001			
Duration of non-group walks	0.01	0.01	0.11	0.004			
Natural & Semi-Natural	0.70	1.12	0.05	0.53			
Green corridor	1.65	1.13	0.10	0.15			
Farmland	2.06	1.22	0.10	0.09			
Urban Green Space	0.96	1.30	0.04	0.46			
Coastal	0.09	1.43	0.003	0.95			
Mixture of 2 or more	1.14	1.48	0.03	0.44			

Note: Step 1: R^2^ = 0.056, Adj R^2^ = 0.052 *p* < 0.001. Step 2: R^2^ = 0.064, Adj R^2^ = 0.052, *p* < 0.001; ∆R^2^ = 0.008 (*p* = 0.403). *B* = regression coefficient, β = standardized regression coefficient. Marital Status: Reference category was single, divorced, widowed. Physical Activity: 0 = 0 days, 7 = 7 days. Duration of non-group walks in green space (in minutes) range was 0 min to 195 min (3 h 15 min). Environment type: Reference category was Urban Public Space.

#### 3.2.5. Negative Affect and Environment Type

In the initial model, a significant 8.5% of the variance in log-transformed negative affect was explained by age, marital status, physical activity and recent stressful life events (see Table 9). The significant negative association for age, marital status and physical activity, and the significant positive association of recent stressful life events remained in the final model. With all predictors in the equation, walk environment type accounted for a significant increase of the variance explained by the model (R^2^ = 0.113, Adjusted R^2^ = 0.100, *p* < 0.001; ∆R^2^ = 0.027, *p* = 0.002). Of the 6 walk environment types, participants who frequently attended WfH group walks in green corridor environments (β = −0.16, *p* = 0.03) and in farmland (β = −0.19, *p* = 0.002) were significantly associated with less negative affect in comparison with participants who frequently attended WfH group walks in urban public space.

**Table 9:** Hierarchical regression analyses for variable predicting log-transformed negative affect for frequent group walkers attending at least once a week (*n* = 708).

Variables	Negative Affect ^				*F*	*df*	*p*
	*B*	*SE B*	β	*p*			
Step 1					16.398	4, 703	<0.001
Constant	1.21	0.02		<0.001			
Age	−0.05	0.02	−0.12	0.002			
Marital Status	−0.03	0.01	−0.12	0.001			
Physical Activity	−0.01	0.003	−0.11	0.002			
Recent stressful life events ^	0.13	0.02	0.21	<0.001			
Step 2					8.857	10, 697	<0.001
Constant	1.23	0.02		<0.001			
Age	−0.04	0.02	−0.09	0.01			
Marital Status	−0.03	0.01	−0.11	0.003			
Physical Activity	−0.01	0.00	−0.12	0.001			
Recent stressful life events ^	0.13	0.02	0.21	<0.001			
Natural & Semi-Natural	−0.03	0.02	−0.12	0.10			
Green corridor	−0.04	0.02	−0.16	0.03			
Farmland	−0.07	0.02	−0.19	0.002			
Urban Green Space	−0.01	0.02	−0.02	0.66			
Coastal	0.01	0.03	0.01	0.84			
Mixture of 2 or more	−0.01	0.03	−0.02	0.63			

Note: Step 1: R^2^ = 0.085, Adj R^2^ = 0.080, *p* < 0.001. Step 2: R^2^ = 0.113, Adj R^2^ = 0.100, *p* < 0.001; ∆R^2^ = 0.027 (*p* = 0.002). *B* = regression coefficient, β = standardized regression coefficient. Age: Reference category was 18–54 years of age. Marital Status: Reference category was single, divorced, widowed. Physical Activity: 0 = 0 days, 7 = 7 days. Recent stressful life events range was 0–11 stressful events. Environment type: Reference category was Urban Public Space. ^ = log-transformed variable.

## 4. Discussion

The focus of the study was to examine whether there was an “added benefit” on psychological and emotional well-being from the type of natural environment in which a group walk took place compared to group walks in urban environments. Participants who attended a Walking for Health group walk at least once a week during the 13-week study period completed an online questionnaire about their mental well-being, depression, perceived stress, positive affect, negative affect and other covariates. Using hierarchical regression and controlling for significant non-environment related covariates (e.g., age, physical activity), we found environment type to be non-significant for mental well-being, depression or positive affect. Environmental type did, however, significantly improve the prediction of less perceived stress and negative affect.

### 4.1. Differences in Well-Being between Group Walks in Specific Types of Environments

The impact of specific types of natural environments on psychological and emotional well-being was also investigated. For depression and positive affect there was no difference between group walks in any type of natural environment compared to group walks in the urban environment. For mental well-being, perceived stress and negative affect there was a significant difference between group walks in farmland and those taken in the urban environment; group walks in farmland were significantly associated with less perceived stress and negative affect, and with greater mental well-being. The results for farmland environments are supported by de Vries *et al*. [57], who found a positive association between the amount of farmland near the home and mental health. Group walks in green corridor environments were also significantly associated with less perceived stress and negative affect, and (marginally) associated with greater mental well-being, when compared to group walks in the urban environment. The results for green corridor environments are consistent with previous studies [60,61] in which levels of mental and emotional well-being were greater in waterside environments (NB: the “green corridor” category included “river path”). No significant effect was found for group walks in natural and semi-natural places, urban green space, coastal, and mixed environments on mental well-being, perceived stress and negative affect, when compared to group walks in the urban environment.

Our findings for perceived stress and negative affect support the psycho-physiological stress reduction framework [34], which posits that nature decreases negative emotion and stress and increases positive emotion. These findings mirror previous research that found a reduction in negative affect and perceived stress from walks in the natural environment [41,42,45] compared to walks in the urban environment. However, our findings did not support theory and previous research for greater positive affect from walks in the natural environment compared to walks in the urban environment [41,42,43,44]. The social environment of the group walk, and the other significant predictors of positive affect may explain this lack of change in positive affect by type of environment.

The lack of differentiation between environment types may be related to the fact that these are group walks, *i.e.*, walking with others may have increased the experience of the urban environment or contracted the experience of the natural environments. Previous research suggests that feelings of restoration from natural environments may be diminished when walking with others. For example, the effects of psychological restoration from a nature setting are greater when alone than with others‒but only if the person feels safe [49]. Johannsson *et al.* [24] found that feelings of revitalization were greater when walking alone in a park compared to walking in a park with a friend. Conversely, feelings of revitalization were greater when walking in an urban environment with a friend compared to walking alone in an urban environment [24]. White *et al*. [55] found that visiting an environment with other adults was associated with significantly less restoration compared to being alone in the environment. Walking with others may change how one interacts with the natural environment, which could influence the mental and emotional well-being benefits from walking outdoors [96]. It is possible that interactions with the natural environment when on a WfH walk are incidental to the walking activity or social interaction. Indeed, qualitative research of WfH group walks suggests that group walkers are more concerned with brisk walking or talking with others than the environment [97].

### 4.2. Dose-Response

The results show an interesting dose-response relationship between outdoor walk behavior and several aspects of well-being. Depression, positive affect and perceived stress demonstrated dose-responses for duration and frequency of walking. Depression showed a (marginally significant) inverse relationship with duration of a group walk, in that depression decreased as a group walk increased by 15 min. Positive affect significantly improved as the duration of non-group walks in green space increased by an additional 15 min. Barton and Pretty [60] similarly found improvements in self-esteem and mood after a short duration of exercise (*i.e.*, 5 min) in the natural environment although other observational studies have found no effect of duration of outdoor exercise on well-being [98,99]. Perceived stress decreased significantly as the frequency of non-group walks in green space increased by one walk. Hamer *et al*. [100] found that higher frequency of walking (once a week or more) was independently associated with a lower risk of mental ill health. The research into frequency or duration of walks outdoors and well-being has been described as “unclear” [40] (p. 1767), as the majority of studies on walking in nature and well-being are experimental cross-over designs with a single bout of exercise for a short, defined period of time [40]. Results from our observational study illustrate the influence frequent walks in green space of variable duration may have on multiple indictors of well-being. Specifically, our results suggest that small changes in walk behavior—such as one extra walk in green space per week or walking for an additional 15 min—could have a positive influence on emotion and stress.

### 4.3. Physical Exercise and Well-Being

Physical exercise was found to have an effect on mental well-being, depression and positive and negative affect. However, our results also suggest an “added benefit” to mental well-being and negative affect from group walks in farmland environments that is above and beyond the effect of physical activity. The impact of physical activity on psychological and emotional well-being is well documented [16]. Previous results have found that walking *per se* was associated with improvements in well-being, irrespective of the type of environment or the social condition for walking [24,25]. Johansson *et al.* [24] found that walking increased positive affect, irrespective of whether it occurred in a park or in urban public space. Mood improved irrespective of whether one was walking alone or with a friend in either a university campus or indoors [25]. Pretty *et al.* [98] found that all types of physical activity outdoors—irrespective of the environment, duration or intensity—increased self-esteem and mood. Barton and Pretty [60] found there were “no great differences” (p. 3949) in self-esteem and mood between exercise in urban space, countryside and woodland environments, suggesting physical activity outdoors was the main cause for change in these measures. Issacs *et al*. [101] found there were no significant differences for anxiety and depression between participants randomly assigned to a walking group, gym exercise or an advice-only control group. Our results, and those of previous literature, highlight the need to control for other physical activity when analyzing the unique contribution of the type of environment for a walk to well-being.

### 4.4. Stressful Life Event and Well-Being

Recently experienced stressful life events were significantly associated with the negative aspects of well-being—depression, perceived stress and negative affect. The negative relationship between stressful life events and well-being is well known [102,103]. However this study found a significant effect of the type of environment for a walking group on perceived stress and negative affect, over and above the effect of recent stressful life events. Specifically, frequent group walks in green corridor and farmland environments were associated with a reduction in perceived stress and negative affect. These results suggest that frequent group walks in these specific environments may be a protective factor against the negative effects of stressful life events on perceived stress and negative affect.

### 4.5. Strengths and Limitations of the Study

The strengths of the study include its relatively large sample of adults from the general population of England. The large sample size enabled comparisons between seven different walk environment types as well as the statistical control of physical activity and other significant predictors of well-being. This study measured various indicators of well-being and their relationship to outdoor walking, thus contributing to the investigation of the effect of natural environments on multiple dimensions of well-being.

The study does have a number of limitations. Firstly, care has to be taken in generalizing beyond the sample. The subsample was restricted to participants who frequently attended a walking group once a week or more. Frequently attending WfH walkers represent a minority of individuals who participate in the WfH program [82]. Moreover, the subsample was mostly female, aged 55 or older, married and living in the least deprived areas of England. As such, the subsample is unlikely to be representative of the adult general population living in England. Furthermore, participants in the study were a self-selected sample of motivated, computer literate individuals who had the time to complete two lengthy questionnaires. Secondly, the use of standardized measures meant that well-being was assessed over a time frame “in the last two weeks”. The analyses sought to relate attending group walks in a certain environment at least twice over the time period to well-being experienced in that same period. There are, however, other events, occurrences or behaviors a participant may/may not have experienced or undertaken on a daily basis that could affect well-being (e.g., physical activity, deprivation of living environment, marital status, stressful life events). The analysis accounted for a few of these potential confounding variables on well-being by including them in the hierarchical regression model. Thirdly, although we controlled for confounding variables, other unmeasured explanatory variables could account for the group differences, such as region of the UK, intensity of group walks in each environment [60], the social aspect of a walking group [24] or personal drivers for participating in outdoors walking groups. Indeed the low overall predictive power of the final model highlights the need to examine additional explanatory variables. Lastly, the observational, cross-sectional design of this study limits conclusions about causality.

### 4.6. Future Research

Future studies could isolate the effects of walking, the social environment and the physical environment. To isolate the effects of walking and the physical environment, an experimental study could randomly assign individuals to a walking group in different types of environments or a wait-list control group. To isolate the effects of the social and physical environments on walks, future studies could compare walking in specific types of environments alone or with a group. Future research could also investigate whether certain types of environments facilitate social well-being better than others. However, such studies would necessitate quite a large sample size in order to have the power to perform the between group comparisons presented here. Future studies may want to consider participant perceptions of the quality of the different types of environments.

### 4.7. Implications

Group walking programs can increase physical activity, which can lower the risk of CVD, obesity [104] and depression [105,106]. The results here could be used to facilitate recruitment to group walking programs. Physical activity decreases as age increases [10], but walking programs appear to reverse this trend. For example, 72% of individuals who participate in the WfH program are aged 55 or over [82]. Participants in this study who were aged 55 or over expressed less negative aspects of well-being, more so than younger participants. This result may have relevance for combating mental ill health in this age group, whilst also increasing physical activity.

Our findings add support for the use of group walking programs for “green prescriptions”. A “green prescription” is an outdoor physical activity prescription from a health care practitioner [107] and are issued in the United States [108], New Zealand [109] and in Scotland [107]. Participants who frequently attended a walking group at least once a week were also frequently participating in other physical activity. An increase in physical activity of frequent group walkers was associated with greater mental well-being and positive affect and less depression and negative affect. We found that outdoor walks of greater frequency and duration were associated with greater positive affect, and less perceived stress and depression. The type of natural environment for a group walk could have an effect on well-being, above and beyond the effects of physical activity. “Green prescriptions” for group walks in green corridor and farmland environments may reduce perceived stress and negative affect of patients.

## 5. Conclusions

Much has been written about the psychological or emotional benefits from interaction with nature [26,27,28,29]. Recent systematic reviews have concluded that there are emotional well-being benefits from engaging in physical activity in natural environments when compared to indoor or urban environments [30,40]. The majority of studies of walking and well-being investigate a participant walking alone in one type of natural environment compared to walking alone in the urban environment or indoors. Results from this study contribute to this research area by showing an effect that walking in a group in different types of natural environments can have on psychological and emotional well-being, when compared to group walks in the urban environment. The benefits of outdoor group walks suggest the importance of such programs for improving psychological and emotional well-being and increasing physical health through physical activity.

## References

[1] World Health Organization Preamble to the Constitution of the World Health Organization as adopted by the International Health Conference, New York, 19 June–22 July 1946; signed on 22 July 1946 by the Representatives of 61 States (Official Records of the World Health Organization, No. 2, p. 100) and Entered into Force on 7 April 1948. The Definition has not been Amended since 1948. http://www.who.int/suggestions/faq/en/.

[2] World Health Organization Cardiovascular Diseases (CVDs) Factsheet Number 317. http://www.who.int/mediacentre/factsheets/fs317/en/.

[3] World Federation for Mental Health Depression: A Global Crisis. World Mental Health Day, October 10 2012. http://www.wfmh.org/2012DOCS/WMHDay%202012%20SMALL%20FILE%20FINAL.pdf.

[4] World Health Organization (2008). 2008–2013 Action Plan for the Global Strategy for the Prevention and Control of Noncommunicable Diseases.

[5] Boone-Heinonen J., Evenson K.R., Taber D.R., Gordon-Larsen P. (2009). Walking for prevention of cardiovascular disease in men and women: A systematic review of observational studies. Obes. Rev..

[6] Morabia A., Costanza M.C. (2004). Does walking 15 minutes a day keep the obesity epidemic away? Simulation of the efficacy of a population wide campaign. Am. J. Public Health.

[7] Pucher J., Buehler R., Bassett D., Dannenberg A. (2010). Walking and cycling to health: A comparative analysis of city, state, and international data. Am. J. Public Health.

[8] Robertson R., Robertson A., Jepson R., Maxwell M. (2012). Walking for depression or depressive symptoms: A systematic review and meta-analysis. Ment. Health Phys. Act..

[9] Armstrong K., Edwards H. (2004). The effectiveness of a pram-walking exercise programme in reducing depressive symptomatology for postnatal women. Int. J. Nurs. Pract..

[10] Craig R., Mindell J., Hirani V. (2009). Health Survey for England 2008: Volume 1 Physical Activity and Fitness.

[11] Centers for Disease Control and Prevention 6 in 10 Adults Now Get Physical Activity by Walking. http://www.cdc.gov/features/vitalsigns/walking/.

[12] Centers for Disease Control and Prevention Facts about Physical Activity. http://www.cdc.gov/physicalactivity/data/facts.html.

[13] Department of Health (2011). Start Active, Stay Active: A Report on Physical Activity for Health from the Four Home Countries’ Chief Medical Officers.

[14] Sallis J.F., Owen N., Fisher E.B., Glanz K., Rimer B.K., Wiswanath K. (2008). Ecological Models of Health Behavior. Health Behavior and Health Education: Theory, Research and Practice.

[15] Ball K., Bauman A., Leslie E., Owen N. (2001). Perceived environmental aesthetics and convenience and company are associated with walking for exercise among Australian adults. Prev. Med..

[16] Biddle S.J.H., Mutrie N. (2008). Psychology of Physical Activity: Determinants, Well-Being and Interventions.

[17] Lee A.C.K., Maheswaran R. (2011). The health benefits of urban green spaces: A review of the evidence. J. Public Health.

[18] Owen N., Humpel N., Leslie E., Bauman A., Sallis J.F. (2004). Understanding environmental influences on walking. Am. J. Prev. Med..

[19] McCormack G.R., Rock M., Toohey A.M., Hignell D. (2010). Characteristics of urban parks associated with park use and physical activity: A review of qualitative research. Health Place.

[20] Kaczynski A.T., Henderson K.A. (2007). Environmental correlates of physical activity: A review of evidence about parks and recreation. Leis. Stud..

[21] Gatersleben B., Andrews M. (2013). When walking in nature is not restorative—The role of prospect and refuge. Health Place.

[22] Mytton O.T., Townsend N., Rutter H., Foster C. (2012). Green space and physical activity: An observational study using health survey for England data. Health Place.

[23] Kwak L., Kremers S., Walsh A., Brug H. (2005). How is your walking group running?. Health Educ..

[24] Johansson M., Hartig T., Staats H. (2011). Psychological benefits of walking: Moderation by company and outdoor environment. Appl. Psychol. Health Well-Being.

[25] Plante T.G., Gores C., Brecht C., Carrow J., Imbs A., Willemsen E. (2007). Does exercise environment enhance the psychological benefits of exercise for women?. Int. J. Stress Manag..

[26] Keniger L.E., Gaston K.J., Irvine K.N., Fuller R.A. (2013). What are the benefits of interacting with nature?. Int. J. Environ. Res. Public Health.

[27] Irvine K., Warber S. (2002). Greening healthcare: Practicing as if the natural environment really mattered. Altern. Therapies. Health Med..

[28] Croucher K., Myers L., Bretherton J. (2007). The Links between Greenspace and Health: A Critical Literature Review.

[29] Pretty J., Barton J., Colbeck I., Hine R., Mouranto S., MacKerron G., Wood C. (2011). Health Values from Ecosystems. The UK National Ecosystem Assessment Technical Report.

[30] Bowler D.E., Buyung-Ali L., Knight T., Pullin A.S. (2010). A systematic review of evidence for the added benefits to health of exposure to natural environments. BMC Public Health.

[31] Kaplan R., Kaplan S. (1989). The Experience of Nature: A Psychological Perspective.

[32] Kaplan S. (1995). The restorative benefits of nature: Toward an integrative framework. J. Environ. Psychol..

[33] Ulrich R., Altman I., Wohlwill J. (1983). Aesthetic and Affective Response to Natural Environment. Human Behavior and the Natural Environment.

[34] Ulrich R., Simons R., Losito B., Fiorito E., Miles M., Zelson M. (1991). Stress recovery during exposure to natural and urban environments. J. Environ. Psychol..

[35] Ulrich R. (1984). View through a window may influence recovery from surgery. Science.

[36] Kaplan R. (2001). The nature of the view from home: Psychological benefits. Environ. Behav..

[37] Kuo F.E. (2001). Coping with poverty—Impacts of environment and attention in the inner city. Environ. Behav..

[38] Parsons R., Tassinary L., Ulrich R., Hebl M., Grossman-Alexander M. (1998). The view from the road: Implications for stress recovery and immunization. J. Environ. Psychol..

[39] Tennessen C., Cimprich B. (1995). Views to nature: Effects on attention. J. Environ. Psychol..

[40] Thompson Coon J., Boddy K., Stein K., Whear R., Barton J., Depledge M.H. (2011). Does participating in physical activity in outdoor natural environments have a greater effect on physical and mental wellbeing than physical activity indoors? A systematic review. Environ. Sci. Technol..

[41] Hartig T., Evans G.W., Jamner L.D., Davis D.S., Garling T. (2003). Tracking restoration in natural and urban field settings. J. Environ. Psychol..

[42] Berman M., Jonides J., Kaplan S. (2008). The cognitive benefits of interacting with nature. Psychol. Sci..

[43] Focht B.C. (2009). Brief walks in outdoor laboratory environments: Effects on affective responses, enjoyment, and intentions to walk for exercise. Res. Q. Exerc. Sport.

[44] Baker G., Gray S.R., Wright A., Fitzsimons C., Nimmo M., Lowry R., Mutrie N. (2008). The effect of a pedometer-based community walking intervention “Walking for Wellbeing in the West” on physical activity levels and health outcomes: A 12-week randomized controlled trial. Int. J. Behav. Nutr. Phys. Act..

[45] Park B., Furuya K., Kasetani T., Takayama N., Kagawa T., Miyazaki Y. (2011). Relationship between psychological responses and physical environments in forest settings. Landsc. Urban Plan..

[46] Peacock J., Hine R., Pretty J. (2007). Got the Blues, then Find Some Greenspace: The Mental Health Benefits of Green Exercise Activities and Green Care.

[47] Barton J., Griffin M., Pretty J. (2012). Exercise-, nature- and socially interactive-based initiatives to improve mood and self-esteem in the clinical population. Perspect. Public Health.

[48] Sugiyama T., Leslie E., Giles-Corti B., Owen N. (2008). Associations of neighbourhood greenness with physical and mental health: Do walking, social coherence and local social interaction explain the relationships?. J. Epidemiol. Community Health.

[49] Staats H., Hartig T. (2004). Alone or with a friend: A social context for psychological restoration and environmental preferences. J. Environ. Psychol..

[50] Velarde M.D., Fry G., Tveit M. (2007). Health effects of viewing landscapes—Landscape types in environmental psychology. Urban For. Urban Green..

[51] Jorgensen A., Gobster P.H. (2010). Shades of green: Measuring the ecology of urban green space in the context of human health and well-being. Nat. Cult..

[52] Mitchell R. (2013). Is physical activity in natural environments better for mental health than physical activity in other environments?. Soc. Sci. Med..

[53] Martens D., Gutscher H., Bauer N. (2011). Walking in “wild” and “tended” urban forests: The impact on psychological well-being. J. Environ. Psychol..

[54] Pretty J., Peacock J., Sellens M., Griffin M. (2005). The mental and physical health outcomes of green exercise. Int. J. Environ. Health Res..

[55] White M.P., Pahl S., Ashbullby K., Herbert S., Depledge M.H. (2013). Feelings of restoration from recent nature visits. J. Environ. Psychol..

[56] Maas J., Verheij R.A., Groenewegen P.P., de Vries S., Spreeuwenberg P. (2006). Green space, urbanity, and health: How strong is the relation?. J. Epidemiol. Community Health.

[57] De Vries S., Verheij R., Groenewegen P., Spreeuwenberg P. (2003). Natural environments-healthy environments? An exploratory analysis of the relationship between greenspace and health. Environ. Plan. A.

[58] Wheeler B.W., White M., Stahl-Timmins W., Depledge M.H. (2012). Does living by the coast improve health and wellbeing?. Health Place.

[59] White M.P., Alcock I., Wheeler B.W., Depledge M.H. (2013). Coastal proximity, health and well-being: Results from a longitudinal panel survey. Health Place.

[60] Barton J., Pretty J. (2010). What is the best dose of nature and green exercise for improving mental health? A multi-study analysis. Environ. Sci. Technol..

[61] Hinds J., Sparks P. (2011). The affective quality of human-natural environment relationships. Evolut. Psychol..

[62] Fuller R., Irvine K., Devine-Wright P., Warren P., Gaston K.J. (2007). Psychological benefits of greenspace increase with biodiversity. Biol. Lett..

[63] Dallimer M., Irvine K.N., Skinner A.M.J., Davies Z.G., Rouquette J.R., Maltby L.L., Warren P.H., Armsworth P.R., Gaston K.J. (2012). Biodiversity and the feel-good factor: Understand associations between self-reports human well-being and species richness. BioScience.

[64] Fitches T. (2011). Who Took Part in Walking for Health? An Analysis of Walker Demographics April 2008 to April 2010.

[65] Walking for Health The case for Walking for Health: A briefing for Scheme Coordinators. http://www.walkingforhealth.org.uk/sites/default/files/caseforsupportbriefing-final.pdf.

[66] Tennant R., Hiller L., Fishwick R., Platt S., Joseph S., Weich S., Parkinson J., Secker J., Stewart-Brown S. (2007). The Warwick-Edinburgh Mental Well-being Scale (WEMWBS): Development and UK validation. Health Qual. Life Outcomes.

[67] HM Government (2011). No Health without Mental Health: A Cross-Government Mental Health Outcomes Strategy for People of all Ages.

[68] Hine R., Wood C., Barton J., Pretty J. (2011). The Mental Health and Wellbeing Effects of a Walking and Outdoor Activity Based Therapy Project. A Report for Discovery Quest and Julian Housing.

[69] Ward Thompson C., Roe J., Aspinall P., Mitchell R., Clow A., Miller D. (2012). More green space is linked to less stress in deprived communities: Evidence from salivary cortisol patterns. Landsc. Urban Plan..

[70] Olsen L., Mortensen E., Bech P. (2004). Prevalence of major depression and stress indicators in the danish general population. Acta Psychiatr. Scand..

[71] Bech P., Rasmussen N.-A., Olsen L.R., Noerholm V., Abildgaard W. (2001). The sensitivity and specificity of the Major Depression Inventory, using the present state examination as the index of diagnostic validity. J. Affect. Disord..

[72] Munir F., Nielsen K., Carneiro I. (2010). Transformational leadership and depressive symptoms: A prospective study. J. Affect. Disord..

[73] Forsell Y. (2005). The major depression inventory *versus* schedules for clinical assessment in neuropsychiatry in a population sample. Soc. Psychiatry Psychiatr. Epidemiol..

[74] Powell J., McCarthy N., Eysenbach G. (2003). Cross-sectional survey of users of internet depression communities. BMC Psychiatry.

[75] Taylor L., Wicks P., Leigh P.N., Goldstein L.H. (2010). Prevalence of depression in amyotrophic lateral sclerosis and other motor disorders. Eur. J. Neurol..

[76] Cohen S., Kamarck T., Mermelstein R. (1983). A global measure of perceived stress. J. Health Soc. Behav..

[77] Stigsdotter U.K., Ekholm O., Schipperijn J., Toftager M., Kamper-Jørgensen F., Randrup T.B. (2010). Health promoting outdoor environments—Associations between green space, and health, health-related quality of life and stress based on a Danish national representative survey. Scand. J. Public Health.

[78] Watson D., Clark L.A., Tellegen A. (1988). Development and validation of brief measures of positive and negative affect: The PANAS scales. J. Personal. Soc. Psychol..

[79] Berman M., Kross E., Krpan K., Askren M., Burson A., Deldin P., Kaplan S., Sherdell L., Gotlib I., Jonides J. (2012). Interacting with nature improves cognition and affect for individuals with depression. J. Affect. Disord..

[80] Van den Berg A.E., Custers M.H.G. (2011). Gardening promotes neuroendocrine and affective restoration from stress. J. Health Psychol..

[81] Crawford J.R., Henry J.D. (2004). The positive and negative affect schedule (PANAS): Construct validity, measurement properties and normative data in a large non-clinical sample. Br. J. Clin. Psychol..

[82] Coleman R.J., Kokolakakis T., Ramchandani G. (2011). Walking for Health Attendance Study.

[83] Office for National Statistics. Analysis of Experimental Subjective Well-being Data from the Annual Population Survey, April to September 2011. http://www.ons.gov.uk/ons/dcp171776_257882.pdf.

[84] OECD Compendium of OECD Well-being Indicators. http://www.oecd.org/general/compendiumofoecdwell-beingindicators.htm.

[85] Steptoe A., Wright C., Kunz-Ebrecht S.R., Iliffe S. (2006). Dispositional optimism and health behaviour in community-dwelling older people: Associations with healthy ageing. Br. J. Health Psychol..

[86] Department for Communities and Local Government The English Indices of Deprivation 2010. https://www.gov.uk/government/uploads/system/uploads/attachment_data/file/6871/1871208.pdf.

[87] Tabachnick B.G., Fidell L.S. (2013). Using Multivariate Statistics.

[88] Milton K., Bull F., Bauman A. (2011). Reliability and validity testing of a single-item physical activity measure. Br. J. Sports Med..

[89] Walking for Health Outdoor Health Questionnaire. http://www.walkingforhealth.org.uk/sites/default/files/OHQ-050313-new-brand-colour.pdf.

[90] Brugha T., Cragg D. (1990). The list of threatening experiences: The reliability and validity of a brief life events questionnaire. Acta Psychiatr. Scand..

[91] Brugha T., Bebbington P., Tennant C., Hurry J. (1985). The list of threatening experiences: A subset of 12 life event categories with considerable long-term contextual threat. Psychol. Med..

[92] Walking for Health (2011). Walk Route Assessment Sheet.

[93] Department for Communities and Local Government (2002). Planning Policy Guidance 17: Planning for Open Space, Sport and Recreation.

[94] White M.P., Smith A., Humphryes K., Pahl S., Snelling D., Depledge M. (2010). Blue space: The importance of water for preference, affect and restorativeness ratings of natural and built scenes. J. Environ. Psychol..

[95] Phillips R., Knox A., Langley E. (2011). Walking for Health: ‘Inactive’ Walkers—Barriers to Participation, and Activity Substitution.

[96] Duvall J. (2010). Enhancing the benefits of outdoor walking with cognitive engagement strategies. J. Environ. Psychol..

[97] Hynds H., Allibone C. (2009). What Motivates People to Participate in Organised Walking Activity?.

[98] Pretty J., Peacock J., Hine R., Sellens M., South N., Griffin M. (2007). Green exercise in the UK countryside: Effects on health, and psychological well-being, and implications for policy and planning. J. Environ. Plan. Manag..

[99] Mackay G.J., Neill J.T. (2010). The effect of “green exercise” on state anxiety and the role of exercise duration, intensity and greenness: A quasi-experimental study. Psychol. Sport Exerc..

[100] Hamer M., Stamatakis E., Steptoe A. (2009). Dose-response relationship between physical activity and mental health: The scottish health survey. Br. J. Sports Med..

[101] Issacs A.J., Critchley J.A., Tai S.S., Buckingham K., Westley D., Harridge S.D.R., Smith C. (2007). Exercise Evaluation Randomised Trial (EXERT): A randomised trial comparing GP referral for leisure-centre based exercise, community-based walking and advice only. Health Technol. Assess..

[102] Cohen S. (2000). Measures of Psychological Stress.

[103] Radloff L.S. (1977). A self-report depression scale for research in the general population. Appl. Psychol. Meas..

[104] Kahn E.B., Ramsey L.T., Brownson R.C., Heath G.W., Howze E.H., Powell K.E., Stone E.J., Rajab M.W., Corso P. (2002). The effectiveness of interventions to increase physical activity: A systematic review. Am. J. Prev. Med..

[105] Priest P. (2007). The healing balm effect: Using a walking group to feel better. J. Health Psychol..

[106] Holmes G., Evans N. Walk and Talk. Proceedings of the 1st International Conference on the Multi-Dimensional Aspects of Wellbeing.

[107] Jepson R., Robertson R., Cameron H. (2010). Green Prescription Schemes: Mapping and Current Practice.

[108] Institute at the Golden Gate Park Prescriptions: Profiles and Resources for Good Health from the Great Outdoors. http://www.parksconservancy.org/assets/programs/igg/pdfs/park-prescriptions-2010.pdf.

[109] New Zealand Ministry of Health How the Green Prescription Works. http://www.health.govt.nz/our-work/preventative-health-wellness/physical-activity/green-prescriptions/how-green-prescription-works.

